# The Janus Face of NKT Cell Function in Autoimmunity and Infectious Diseases

**DOI:** 10.3390/ijms19020440

**Published:** 2018-02-01

**Authors:** Alessandra Torina, Giuliana Guggino, Marco Pio La Manna, Guido Sireci

**Affiliations:** 1 Experimental Zooprophylactic Institute of Sicily, Via Marinuzzi 3, 90100 Palermo, Italy; alessandra.torina@izssicilia.it; 2 Biomedical Department of Internal and Specialized Medicine, Rheumatology Section, University of Palermo, Piazza delle Cliniche 2, 90100 Palermo, Italy; giuliana.guggino@unipa.it; 3 Department of Biopathology and Medical Biotechnology, Section of General Pathology, University of Palermo, Via del Vespro 129, 90100 Palermo, Italy; marcopio.lamanna@unipa.it; 4 Central Laboratory Advanced Diagnostic and Biological Research, University Hospital, Via del Vespro 129, 90100 Palermo, Italy

**Keywords:** microbes, autoimmunity, glycolipids, alpha-galactosylceramide, sulfatide, CD1d, NKT

## Abstract

Natural killer T cells (NKT) are a subset of T lymphocytes bridging innate and adaptive immunity. These cells recognize self and microbial glycolipids bound to non-polymorphic and highly conserved CD1d molecules. Three NKT cell subsets, type I, II, and NKT-like expressing different antigen receptors (TCR) were described and TCR activation promotes intracellular events leading to specific functional activities. NKT can exhibit different functions depending on the secretion of soluble molecules and the interaction with other cell types. NKT cells act as regulatory cells in the defense against infections but, on the other hand, their effector functions can be involved in the pathogenesis of several inflammatory disorders due to their exposure to different microbial or self-antigens, respectively. A deep understanding of the biology and functions of type I, II, and NKT-like cells as well as their interplay with cell types acting in innate (neuthrophils, innate lymphoid cells, machrophages, and dendritic cells) and adaptive immunity (CD4^+^,CD8^+^, and double negative T cells) should be important to design potential immunotherapies for infectious and autoimmune diseases.

## 1. Distinctive Functional Activities of Types of NKT

CD1 molecule is a family of glycoproteins expressed on the surface of several antigen-presenting cells (APC) involved in the presentation of glycolipid antigens to T cells [1]. Glycolipids bound to CD1 molecules can generate different types of antigen recognition. Two groups of CD1 molecules were identified depending on their lipid anchoring as described below: (i) CD1a, CD1b, and CD1c expressed on dendritic cells, B cells, and macrophages; (ii) CD1d is mainly expressed on the same APCs of the other forms of CD1. CD1e, an intermediate isoform, is located in the cells and its role is still unclear.

In humans, CD1a–c isoforms are able to bound mycobacterial as well as self-antigens [2,3,4,5,6,7,8,9]. CD1d isoforms activate the majority of NKT cells expressing an invariant T-cell receptor (TCR)-α chain rearrangement and are called type I NKT or invariant NKT (iNKT). CD1a isoforms migrate from endoplasmic reticulum (ER) to cell surface to bind antigens while CD1b, c and d are recycling from ER to membrane and vice versa [3,4,5,6,7,8]. APC expressing CD1d are widely expressed on different type of cells: dendritic cells, macrophages, monocytes, cortical thymocytes. CD1d presenting glycolipid activate type I NKT.

Antigen presenting cells displaying the non-classical histocompatibility molecules (CD1 and MR1) bind glycolipids or vitamin B metabolites; the complex CD1-glycolipids activate NKT cells while MR1-vitamin B metabolites metabolites are recognized by mucosal associated invariant T (MAIT) cells, another subset of T cells showing innate and adaptive features. TCRs involved in recognition of CD1-glycolipids or MR1-vitamin B metabolites complexes have a common distinctive characteristic: they display an invariant α chain and few β chains. In contrast to their reduced antigen receptor repertoire, these cells show a marked plasticity in their functions as demonstrated by the production of different cytokines after in vivo stimulation of naïve mice with α-galactosylceramide (α-GalCer) [10]. A small percentage of NKT produce IL10 in human unstimulated peripheral blood mononuclear cells (PBMC), confirming their immunomodulatory feature [11].

Type I NKT cells use TCR constituted by few β chains pairing with Vα14Jα18 in mice and Vα24Jα18 TCR in humans. They were characterized by the ability to induce strong cytotoxic immune response in a murine cancer model [12]. Type I NKT cells recognize a glycolipid obtained by a marine sponge, α-GalCer, in humans and mice.

Another subset of NKT cells, called type II NKT cells, does not react with α-GalCer, but binds a self-lipid, sulfatide, highly expressed central nervous system (CNS), kidney, pancreas, and liver [13]. They recognize several self-lipids using oligoclonal TCRs expressing Vα3 or Vα1 and Vβ8.1 or Vβ3.1. Type II NKT cells can accumulate in the CNS, suggesting their compartmentalization in this tissue respect to type I NKT (3%/0.6%, respectively) as this tissue displays high expression of sulfatide.

NKT-like cells are another subset able to express constitutively either T cell receptors (TCR) or NK markers (CD16, CD56, CD161) and they were shown to be involved in pulmonary disease [14].

A promising role in adoptive immunotherapies of cancer was assigned to another subset of cells called cytokine-induced killer (CIK) cells [15]. This subset could be obtained by culturing PBMC with anti CD3 beads plus IFN-γ and high doses of IL-2. They comprise lymphocytes with different phenotypes: CD3^+^CD56^+^, CD3^+^CD56^−^, CD3^−^CD56^+^ but they are CD16^−^. Cytokine Induced Killer (CIK) cells are a mixture of NKT-like and NK-like cells. These cells are strong cytotoxic subset whose targets are a wide array of tumors and the mechanism of cytolisis is MHC- or non-MHC-restricted. They do not exert antibody dependent cell cytotoxicity (ADCC) because they lost CD16.

Type I and II NKT can be involved in autoimmune and infectious diseases.

## 2. Type I NKT in Response to Microbial Antigens

Vα14- or Vα24-driven NKT cell response may either promote or inhibit immune response to many different microbial pathogens. Type I NKT driven protection from microbial antigens was analyzed by different authors [16,17,18]. Even if type I NKT expand during various types of infections [16], it was found that the activation of type I NKT by microbial antigens seems to be due at least to two different mechanisms: (i) direct binding of microbial antigens to TCR of type I NKT (direct recognition [19,20]); (ii) type I NKT expansion mediated by cytokines (IL12-IL18) released by other cells (antigen presenting cells like dendritic cells, NK, T cells) during infections (indirect recognition [21,22]). In particular, the indirect recognition, mainly due to IL-12 driven activation of microbial structures by type I NKT, was described not only in bacterial infections (in LPS induced activation [21,22]) or other infectious diseases [23,24,25,26] but also during viral infections and type I NKT activation in virus infected mice seems to be due to an indirect (IL-12-driven) mode of activation [23,27].

α-GalCer, the exogenous ligand of type I NKT, was characterized as a glycosphingolipid able to activate type I NKT. There are microbial cell wall antigens that have same chemical structure of α-GalCer. These glycosphingolipids were described in cell wall of Gram-negative LPS-free *Sphingomonas* species, *S. Yanoiuyakey*. These bacteria are not pathogenic but type I NKT KO mice are exerting a defective clearance of these microbes. Another type of ligand for type I NKT TCR was described in *Borrelia burgdorferi*, a microbe causing Lyme disease. Vα14 KO mice also manifest a defect of clearance of *Borrelia burgdorferi* and after one week of infection NKT are producing IFN-γ and IL-4 [28,29]. *B. burgdorferi* does not display glycosphingolipids but glycosilated diacylglicerol [30,31] that are weak type I NKT ligands.

*Helicobacter pylori*, the causative agent bacteria of gastritis and peptic ulcers, has cholesteryl phosphatidyl α-glucoside. Vα14 knock out (KO) mice have a defective clearance of *H. pylori* but there is not evidence that cholesteryl phosphatidyl α-glucoside could bind to CD1d [32]. Another microbial source of type I NKT antigens is derived from *Entamoeba hystolitica*, a pathogen causing abscesses in the gut. It was found a lipopeptidophosphoglycan derived from *E. hystolitica* that is able to activate iNKT and this event decrease abscesses due to the infection [33].

Another interesting observation about type I NKT response in experimental infectious disease describes an early increase of NKT producing IL17 during *Rickettsia conorii* murine infection. The increase of type I NKT IL17^+^ was detected after three days of infection either ex vivo or after in vitro α-GalCer stimulation [34]. In the same study, we report an early increase of NK IFN-γ^+^ ex vivo, suggesting a cytokine milieu, rich of IL12, derived from dendritic cells (DC), and IFN-γ from NK, that could favor an increase of type I NKT producing IL17 that could be responsible of vasculitis, a pathological feature not only during *Rickettsia* spp. infections but also occurring in autoimmune disorders [35].

A novel mechanism of indirect activation of type I NKT was found in an experimental model of infection by *Leishmania mexicana* [36]. Lipophosphoglycan (LPG), derived from this pathogen, stimulating Toll-like receptor 2 (TLR2) on the membrane of DC, upregulate MHC Class II, B7 and IL-12. These effects cause an increase of IFN-γ by type I NKT and *L. mexicana* lesions were decreased in the mice. A different pathway of activation of type I NKT (direct) was detected in *Leishmania donovani* infection [37]. In this model lipophosphoglycan, obtained from the parasite, bind CD1d, and stimulate TCR of type I NKT.

A direct mechanism of activation of iNKT was reported using a molecule derived from a fungus. A glycosphingolipid, asperamide B, obtained by *Aspergillus fumigatus*, a saprophytic fungus causing allergic disorders in humans, bound by CD1d, activate iNKT cells in an IL33-ST2 pathway, causing allergy [38].

All these studies describe different pathways by which microbes could activate type I NKT subset. Many microbial molecules are able to bind type I NKT TCR directly or these antigens could promote the release of cytokines that induce type I NKT immune responses (indirect pathway of activation). This type of host immune response may exacerbate or protect the host from infections.

## 3. Role of Type II NKT in Immune Responses to Different Microorganisms

Sulfatide-reacting NKT cells (type II NKT) were shown to exert different effects in experimental infectious diseases. In fact, in *Trypanosoma cruzi*-infected mice a proinflammatory effect by type II NKT was described [39] while an opposite effect was described in *Schistosoma mansoni* infection accompanied by secretion of Th2 cytokines was exerted by the same subset [40]. A reduced secretion of TNF-α and IL-6, due to type II NKT activation in *Staphylococcus aureus*-induced sepsis, protected mice from death [41]. It was shown that glycolipids obtained from *Mycobacterium tuberculosis* or *Corynebacterium glutamicum* [42] and phosphatidylglycerol from *Listeria monocytogenes* [43] could activate type II NKT cells.

Controversial effects of type II NKT activation were reported in experimental viral infections. In an experimental model of hepatitis B virus (HBV) infection an activation of type II NKT due to NKG2d cause damage to the liver. In particular, phosphatydiletanolamine and lysophosphatydiletanolamine ER-self lipids obtained by HBV infection induce liver type II NKT activation that transactivate type I NKT cells during infection [44]. Sulfatide-induced type II NKT activation occurring in SCID-hu lymphopoiesis was shown to induce type I NKT anergy during HIV infection [45].

## 4. Type I NKT in Autoimmune and Chronic Inflammatory Diseases

Since NKT can be either pathogenic or protective, studies tried to better define the role of NKT subsets and particularly type I NKT cells appear to have a greater propensity to be more pathogenic than protective but it should be not perfectly applicable in autoimmune and chronic inflammatory disorders. Type I NKT seems to have a role in the regulation of chronic inflammation supporting many autoimmune diseases such as systemic lupus erythematosus (SLE) [46], rheumatoid arthritis (RA) [47], and Sjogren syndrome (SS) [48]. Despite their “classical” pathogenic role in many of these diseases type I NKT cells can display a protective feature.

Reduced numbers of type I NKT cells among PBMC appear to correlate with several autoimmune or inflammatory conditions, together with a possible increase at the anatomical site of inflammation. The reasons for this reduction and compartmentalization, respectively, could be linked in part to differences in the patterns of motility and recirculation of different NKT cells in the blood and target tissues.

A perfect model showing the complex role (protective versus pathogenic) was found in SLE patients. In these patients, type I NKT quantitative deficiency appear to correlate with the activity of SLE disease [46], and these observation is supported from data obtained in lupus prone animal model [49], where, additionally, a lower rate of proliferation to α-GalCer was detected. These results were also confirmed in SLE patients with active disease [50,51]. In vitro studies have demonstrated a defective response of type I NKT from SLE patients to α-GalCer that could be exacerbated by the compromised expression of costimulatory molecule (CD26 [52]). Impaired activation could also influence the cytokine production and in turn contribute to the progression of SLE. On the other hand, other studies have indicated that iNKT cells can secrete IL-17 and other cytokines in several inflammatory diseases, including SLE, depending on the pro-inflammatory environment occurring in damaged tissues [53,54]. These results clarified that type I NKT were complex and pleiotropic. At the same time, the protective role of increase of type I NKT in autoimmunity could be due to the suppressive effects of this subset on autoantibodies production [55]; type I NKT can inhibit CD1d^+^ autoreactive B cells in producing autoantibodies [56]. Another interesting observation of the effect of type I NKT activation on autoimmunity reported protection in an autoimmune experimental model of lupus due to a short term in vivo activation by α-GalCer increasing a subset of IL-10 producing B cells that could inhibit autoantibody secretion [57]. The short term in vivo activation of type I NKT by α-GalCer derivative is able to induce a tolerogenic state, due to anergy of DC and type I NKT, that cause protection of NOD mice by type I diabetes [58].

**Figure 1:**
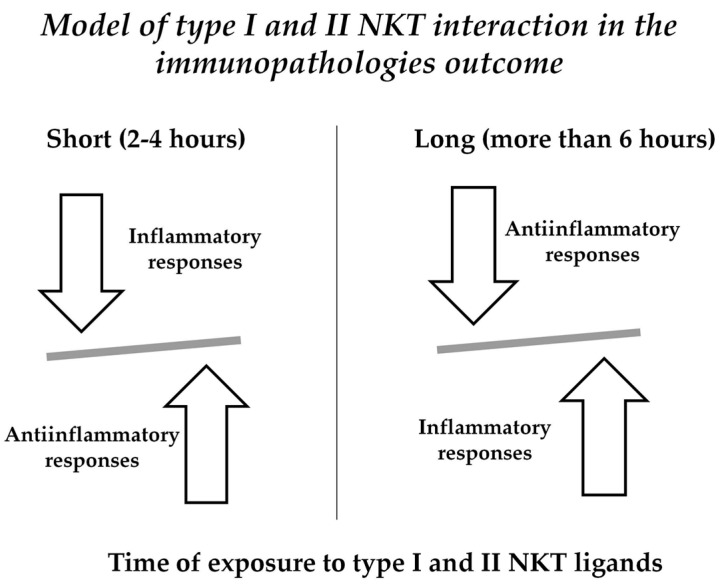
Time-dependent activation of NKT.

We could hypothesize a time-dependent type I and II NKT activation that could modulate inflammation occurring in autoimmunity as it happens in short-term α-GalCer in vivo exposure in naive mice [1] (Figure 1). Moreover, several studies [47,54]—including patients with RA—showed that NKT cells can affect the differentiation of Th cells, including Th1, Th2, Th17, and Treg, via the production of cytokines or cell contact, suggesting an indirect role of NKT cells, boosting the differentiation of CD4^+^ T lymphocytes.

Different types of cytokines are produced depending by the time of exposure of NKT to ligands. Short term activation results in the prevalence of anti-inflammatory molecules (i.e., IL10); pro-inflammatory cytokines (i.e., IFN-γ) are increased in long term (more than 6 h) activation by NKT ligands.

## 5. Type II NKT in Autoimmune and Chronic Inflammatory Diseases

Sulfatide-reacting NKT cells were described initially in central nervous system (SNC) where they are more abundant than type I NKT as sulfatide is abundant in this tissue [59]. Interestingly, in vivo administration of brain-derived or synthetic sulfatide compounds prevent the onset of experimental allergic encephalomyelitis (EAE) and diabetes in non-obese diabetic (NOD) mice [59,60,61]. It was reported that type II NKT, activated by sulfatide, induce anergy of type I NKT and dendritic cells (DC) in EAE [61].

An opposite role in development was described in ulcerative colitis [62,63,64]; in these studies, in humans and mice, type II NKT secreting IL13 in response to lyso-sulfatide are increased [62,63,64] and contribute to inflammation.

Thus, type II NKT may display both protective and pro-inflammatory features and these functions seem to be due to the different types of tissue-specific ligands: tolerogenic molecules in the SNC and pancreas, and inflammatory ligands in the gut.

## 6. CIK Cells as Players of Antimicrobial Immune Response

This hybrid subset of cytotoxic cells, having phenotypes and functional characteristic similar to NKT-like and NK-like subsets, are able to lyse not only many tumors but also other target cells infected by microbes [65]. Cytomegalovirus (CMV) and Epstein–Barr virus (EBV) specific effector memory CD8^+^ T cells are expanded in CIK cultures obtained by PBMC. Interestingly, CIK could be able to kill either virus infected cells or neoplastic cells. It could have a useful application in the immunotherapies in bone marrow transplanted patients. In these cases, CIK infusions could help to eliminate residual leukemic cells and improve the immune response against CMV, EBV, or other microbial infections that could frequently cause severe problems in these type of patients. To this end, this type of intervention has feasibility in fact the numbers of CIK cells obtained from small amounts of blood could justify this kind of helpful strategies. As it was shown that CIK cytolysis could be mediated by NKG2D-dependent mechanism [66], CIK could be active in killing mycobacterial infected cells [67] as well as target cells infected by other pathogens expressing NKG2D.

## 7. Concluding Remarks

**Figure 2:**
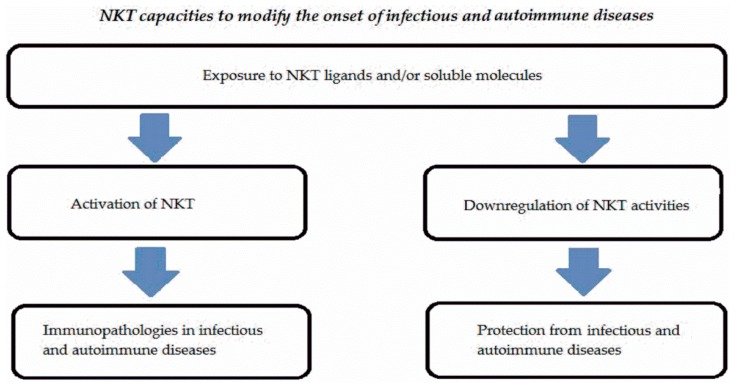
Schematic mechanisms of interaction of NKT in infectious and autoimmune diseases.

NKT cells represent a subset expressed in low percentages in peripheral blood and tissues in humans and mice. These cells are activated by endogenous or exogenous ligands linked to non-polymorphic CD1 molecules and significantly contribute to the onset of infectious or autoimmune diseases. Either type I or type II NKT cells are involved in many infectious or autoimmune disorders. NKT cells may display multiple functions representing a complex system. Figure 2 summarizes the different activities of NKT cells in infectious and autoimmune diseases.

Exposure to NKT ligands expressed by microbes or anatomical districts in combination with cytokine milieu could provide promotional or protective effects for these immunopathologies due not only to NKT activities but also interaction of these cells with other cells (dendritic cells, neutrophils, machrophages, etc.).

Self-reactivity of NKT cells may be due to an evolutionary aspect and could be one of the early links between the innate and adaptive immune systems as a way to respond to various antigens, regardless of their source, that could compromise the integrity of the organism’s tissues. Responding to microbial antigens, NKT cells could have evolved to sense when to limit inflammation to prevent self-tissue destruction, a role consistent with their ability to ameliorate a number of autoimmune conditions as we discussed in this paper [57,58]. The rapid immune response elicited by microbial antigens may be seen as a way for the body to protect itself against damage, a function perhaps coopted into the ability of NKT cells to protect the self even when foreign antigens are not present. A common mechanism by which NKT could act in autoimmunity and microbial infection was reported by De Libero et al. [68]. They report that bacterial infections could promote reaction of NKT to self glycosphingolipids that could induce autoreactivity. Other common mechanisms by which NKT could react to microbes as well as autoantigens could be due to superantigens expressed by bacteria that induce polyclonal activation of T cells responsible for autoimmune responses [69] and innate immune response of NKT could initiate and/or promote the inflammatory status by which an autoimmune disease begins [70].

Binding α-GalCer or sulfatide, type I and II NKT secrete cytokines and/or chemokines and their activation can contribute to the onset of several diseases and could modify the outcome of infections and autoimmune disorders. Soluble factors secreted by NKT cells could act modulating directly or indirectly, transactivating other immune cells (NK, machrophages, DCs, neutrophils, B and T cells, etc.) and promoting a cascade of events with an immunopathogenic roles. CIK, with its hybrid phenotype, could display different types of action of the previous reported cytotoxic T cells, being studied mainly in antitumor immunity but having promising roles in antimicrobial immune response.

Hypothetically, NKT expanded from PBMC of patients exposed for a few hours to α-GalCer could induce anti-inflammatory cytokines (IL10), as previously reported [57,58], downregulating polyclonal activation of T and B cells and related symptoms in autoimmune diseases. CIK cells from patients affected by autoimmune diseases could be transfected with TCR-recognizing autoantigens and injected in patients; they could kill autoreactive cells reacting ameliorating clinical outcome of autoimmune diseases.

The plasticity of NKT and cytotoxic activity of cells could be considered a weapon to build specific immunotherapies.
